# COVID-19 and Internet Hospital Development in China

**DOI:** 10.3390/epidemiologia3020021

**Published:** 2022-06-02

**Authors:** Yushan Li, Huimin Hu, Liudmila Rozanova, Guilhem Fabre

**Affiliations:** 1 Graduate Institute of International and Development Studies (IHEID), Chemin Eugène-Rigot 2, CH-1202 Geneva, Switzerland; 2 Geneva School of Social Sciences, University of Geneva, Uni Mail, Boulevard du Pont-d’Arve 40, CH-1205 Geneva, Switzerland; huimin.hu@etu.unige.ch; 3 Institute of Global Health, University of Geneva, 9 Chemin des Mines, CH-1202 Geneva, Switzerland; liudmila.rozanova@unige.ch; 4 Department of Chinese, UFR 2, Université Paul Valéry Montpellier 3, 34199 Montpellier, France; guilhem.fabre@univ-montp3.fr

**Keywords:** China, COVID-19, digital technology, Health Code, Internet plus Healthcare, Internet hospital

## Abstract

Since 2018, the access policy for Internet hospitals has been issued in China. So far, thousands of Internet hospitals have been approved to operate and have played a significant role during the COVID-19 pandemic. While front-line hospitals strive to treat patients, Internet hospitals take the responsibility to guide patients to seek appropriate medical treatment and meet the urgent needs of chronic patients through online medical follow-up, payment, and drug distribution. This paper is based on Internet medical policies and interviews with doctors working with Internet hospitals, aiming to study the development of Internet hospitals in China through the management of the COVID-19 outbreak and the Chinese healthcare strategy on the national level.

## 1. Introduction

Globally, the public health needs of the population have varied at different times of the pandemic, resulting in other pressures on hospitals. In the pandemic’s early stages, mainly medical conditions and health problems exacerbated by panic put pressure on hospitals. Over time, the consequences of the decline in the availability of medical services caused by large-scale suspension have gradually become apparent, and the crowded medical needs must be met through mediation. Increasing digitization and the application of the Internet have changed the methods people use to search for health information and the way they seek medical services [1]. Online medical services improve the accessibility of quality medical resources and play a role in redistributing medical resources and transforming the structure of the medical system.

China has begun to promote this new model of online healthcare and is actively developing Internet health services. To maximize the potential of the Internet, the Chinese government has developed “Internet plus” concepts to transform, modernize, and equip traditional industries. The healthcare industry has also been combined with the Internet to create “Internet plus Healthcare” (IPHC). Under the influence of COVID-19, the medical habits of users have significantly changed. During the pandemic, Internet hospitals attracted a large number of patients. However, Internet hospitals must meet the absolute need for medical services and provide substantial results rather than just soothing feelings and answering doubts.

The potential significance of this research lies in the comparison of Internet hospitals before and during COVID-19 in terms of their impact on the Chinese healthcare strategy. Another question to explore is the future use of Internet hospitals in healthcare.

## 2. Materials and Methods

Several methods were used to conduct this research, including a literature review, reviewing government documents, and conducting semi-structured interviews with doctors working with Internet hospitals. The following search terms were used to search the Cochrane Library (cochranelibrary.com (accessed on 1 December 2021)), the Medical Literature Analysis and Online Retrieval System (Medline, pubmed.ncbi.nlm.nih.gov), the Medical Abstracts Database (EMBASE, embase.com (accessed on 1 December 2021)), and Nursing and Allied Health Journal Indexes (CINAHL, ebsco.com (accessed on 1 December 2021)) from January 2019 to October 2021: “SARS-CoV-2”, “COVID-19”, “telemedicine”, “telehealth”, “internet hospital”, “ China + epidemiology”, and “non-pharmaceutical intervention”.

An extensive desk review of the literature on COVID-19 epidemiology, non-pharmaceutical intervention (NPI), Internet hospitals, and the Chinese context was carried out. In terms of sources, we used the reports and policy briefs (in English and French) of several international organizations (WHO, ITU, UNICEF) that covered the impact of COVID-19 on the health system of countries and the role, strengths, and weaknesses of telemedicine during COVID-19, which provided the best overview of the pandemic. Official government websites (including China’s State Council, China Health Commission, and China Medical Security Bureau) and documents (in Chinese) were also used because they provide China-specific Internet medical profiles and the latest policies. We also used academic journals and news sources because they tend to offer a more critical assessment of government and health sector responses to COVID-19 impacts and are more likely to discuss an effect that has not had much coverage. We did not collect or analyze policies or interventions that focus on social recovery, which go far beyond the healthcare sector and do not match our study purpose. Overall, although we used some primary sources, most of the references were secondary due to the nature of the research (desk research) for this specific part of the literature review.

We conducted a series of semi-structured interviews with doctors working with Internet hospitals from different regions (Table A1). First, we reached out to doctors in China through our networks and LinkedIn. As a result, in this work, we include the expert opinions of 3 doctors working in Chinese hospitals, specializing in Chinese traditional therapy, neurosurgery, and gastroenterology.

## 3. Results

### 3.1. Health Care System in China: Organization and Governance

Economic outlook: China’s economic growth rate was 2.3% in 2020. Forecasts by the IMF published in October 2021 expect a GDP growth rate of 8% for 2021 [2], supported by the continuous economic transition to high-tech and service-focused industries while challenged by the U.S.–China trade war. Economic growth in the next year will continue to slow down, from 6.6% in 2018 to an estimated 5.0% in 2023. However, government policy support will feed into the economy, including infrastructure investment and targeted looser monetary and fiscal policy. The government is also stimulating private consumption via personal tax reduction to boost domestic needs, leading to increased total spending on healthcare.

Political stability: The current president’s first five years in power culminated in a political triumph in 2017–2018 when he was reappointed to his leadership positions without a designated successor emerging for the handover of power in 2022–2023. This will ensure that President Xi retains authority during his second term. The revision of the national constitution in March 2018 to remove a two-term limit on the offices of the president and vice-president confirmed President Xi’s intention to remain in power for more than the usual decade. Risks to political stability from social upheaval are likely to prove manageable. The strengthening of already tight security procedures, surveillance, and controls on media and the Internet will bolster the Chinese Communist Party (CCP)’s position.

Population health status: The aging population will be the key challenge for China’s population healthcare growth, partially driven by increased life expectancy and partly owing to a decline in the fertility rate. The proportion of the population aged over 65 will continue to grow and is forecast to reach 14.1% by 2023, up from 12.7% in 2018 [3]. The government has initiated a series of policies to meet healthcare needs, from a general approach to improve healthcare service quality and reshuffle central government structure to specific policy to develop chest pain centers and standardize cardiovascular disease treatment SOP.

Health coverage and insurance trends: Government investments in 2012–2020 already extended public reimbursement coverage to rural and unemployed populations. With a compound annual insurance premium growth rate of over 30% for six consecutive years, RMB 817.3 billion worth of premium recorded for 2020, and RMB 2 trillion anticipated for 2025, the health insurance market in China continues to outpace all other lines [3]. The extended fund expects to slow down in the next few years due to high population coverage.

**Figure 1:**
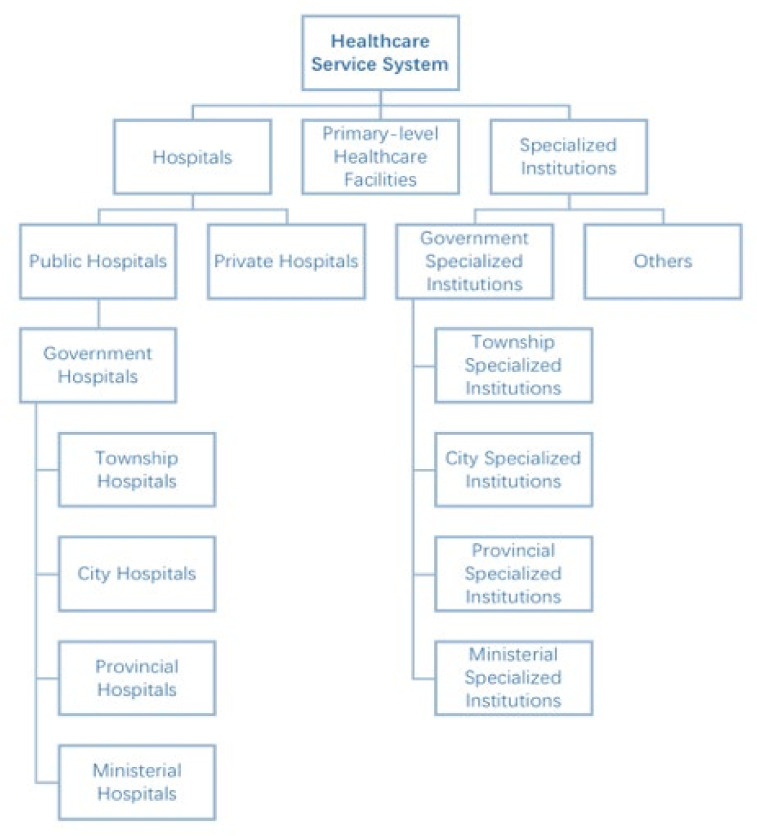
Organization of the healthcare service system in China. (Adapted from source: NHC data [5]).

Healthcare infrastructure development (Figure 1): The hospitals in China are categorized into three tiers based on function, equipment, and technology. The primary hospitals include primary healthcare centers and clinics, which provide preventive care and rehabilitation services for a single community; the secondary hospitals provide medical and health services to multiple communities and undertake specific teaching and scientific research tasks; the tertiary hospitals are medical centers with comprehensive medical, education, and scientific research capabilities and provide high-level specialist care across regions, cities, and provinces [4]. The pandemic of major infectious diseases may bring about profound changes in the public health system. After the outbreak of SARS in 2003, the government increased its determination and action to build a public health system.

China’s health system is also facing the pressure of rising costs. One of the main problems is undoubtedly the uneven distribution of medical and health resources. The reason is that China has vast land and a large population, and many people are distributed in rural areas. In China, only 8% of hospitals are tertiary hospitals, mainly located in big cities and handle up to 50% of outpatients, which also confirms the severity of uneven resource distribution. Most resources are located in large cities and rarely cover rural areas [3,5]. Variable resource allocation leads to long-distance travel for some patients, overcrowding in hospitals in central urban areas, and generally long waiting times. In particular, the problem of long-distance travel is becoming more and more serious, resulting in traffic congestion in big cities. According to the allocation of medical resources in different regions, the construction strategies of health informatization are different [4]. For the developed areas with rich medical resources (mainly the eastern coastal areas), the local health administrative departments bear the primary responsibility of integrating high-quality medical resources and promoting health information. These regions have a strong ability to independently develop informatization, which provides stable and sufficient funds for informatization. Medical resources are scarce in growing areas, mainly in Western and Central China. Large hospitals are usually connected with many local primary healthcare institutions to realize medical information exchange and sharing [6].

Healthcare spending/reimbursement: China has been making efforts to expand medical care coverage, reduce costs, and improve the quality. The National Healthcare Security Administration (NHSA) manages the public medical insurance fund and formulates various public medical insurance policies and processes, such as reimbursement catalog, payment standards and expenses, bidding and procurement process of drugs and medical consumables, etc. The public medical insurance fund managed by NHSA is the largest single payer of China’s medical and health expenditure, accounting for more than 30% of China’s medical and health spending in 2020. In 2020, more than 1.3 billion people in China participated in public health insurance, accounting for more than 95% of the total population [3,5]. With the increasing aging of China’s population, the expenditure pressure of the public medical insurance fund increases. Without effective fund management and a diversified payment structure, the general medical insurance expenditure growth rate will continue to be higher than its income growth. At the same time, China’s commercial health insurance industry is still in the early stage of development with a low penetration rate. China’s per capita commercial health insurance premium is significantly lower than major developed countries.

Regulations: A new government structure was introduced during the State Council reshuffle in March 2018, and a bureaucracy cut was observed in the new system. Three recent government bodies have been established: the National Health Commission (NHC), the Medical Insurance Administration (MIA), and the Administration for Market Regulation (AMR). The reshuffle will allocate more resources to support elder care, diagnostics needs, and chronic disease management; promote local players and produce product development; and promote the development of digitalization in hospitals.

Since the pandemic outbreak, NHSA has issued a series of policies. Special funds were allocated to medical institutions treating patients. Medical treatment in other places is not limited by regulations; adjusting the total medical insurance budget indicators and prepaid medical insurance funds of relevant hospitals ensures that the hospital will not treat patients due to the medical insurance payment policy.

The "Guiding Opinions on the Provision and Reimbursement of Online Health Services during COVID-19 Outbreak”, issued on 2 March 2020, make it clear that patients with chronic diseases can apply for medical insurance reimbursement in Internet hospitals while encouraging the medical institutions to provide online drug purchase services [5]. However, online medical insurance payment for prescription drugs is still limited to the cities the medical insurance belongs to, and it cannot be settled cross-regionally.

### 3.2. COVID-19 in China

#### 3.2.1. Epidemiological Situation in China

It can be seen from historical data that large-scale infectious diseases will lead to considerable morbidity and mortality. According to WHO statistics, as of 14 November 2021, COVID-19 has caused more than 120,000 human infections in China, with a mortality rate of 5% [7].

**Figure 2:**
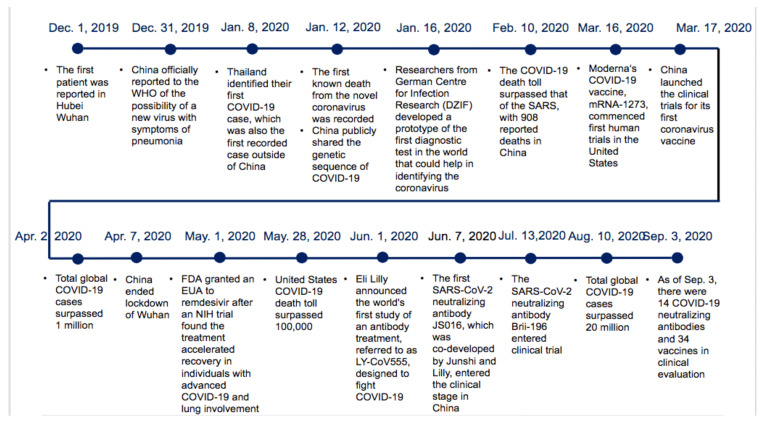
Timeline of the COVID-19 situation in China. (Adapted from source: NHSA data [9]).

China underwent the COVID-19 pandemic in late 2019, which intensified on the eve of the Chinese Lunar Year (Figure 2). The COVID-19 case reporting system was launched by the Chinese government, which took 50 days, compared to the 138 days needed for the severe acute respiratory syndrome (SARS) case reporting system. This progress is due to continuous efforts over the past 17 years, enabling Chinese scientists to quickly identify viruses and share the genome sequencing data of viruses with international researchers [8]. However, China’s public health emergency management is still insufficient. For example, the China Center for Disease Control and Prevention (China CDC) has difficulties recruiting qualified professionals, which significantly restricts its actionability. The lack of laws, regulations, and policies is another crucial factor influencing China’s public health system.

#### 3.2.2. Non-Pharmaceutical Intervention Measures

China’s choice to stick to its Zero-COVID strategy was made possible by maintaining and enforcing NPI measures [10]. NPIs used in China include mask wearing, hand washing, social distancing, isolation of ill individuals, contact tracing, quarantine of exposed individuals, curfews, travel restrictions, and complete lockdowns of selected areas. Digital technology is essential both in the enforcement and mitigation of these measures.

One of the primary uses of digital technology is the Health Code [11,12]. Launched first by the online payment platform Alipay on 9 February 2020, and soon followed by the messaging platform WeChat, this code uses different data sources to indicate a person’s daily updated risk status of having the virus, translated by a color code: green for low risk, yellow for medium risk, and red for high risk. Data used include geolocation data provided by smartphones’ GPS, personal information with the national ID number, and daily updated physical condition (fever, cough, etc.) provided by the individual. The platform requires registration with facial recognition. According to the color, people can access different areas and activities (green) or self-quarantine (yellow and red). For example, a green code is necessary to use public transport, access schools, public buildings, grocery stores, airports, restaurants, and hotels. By August 2020, it had more than 900 million users in over 300 Chinese cities [13]. Furthermore, drones using AI and face recognition allow monitoring people in real time across the country [14].

Although the aim of NPIs is better health through infection control, they also have a negative impact on health, particularly healthcare access. Under travel restrictions, it can be challenging to access adapted healthcare facilities, and isolated or quarantined individuals should not leave their facility but might require medical assistance. This is where digital technology can provide solutions: telemedicine can be accessed without leaving home [11]. Telemedicine, including Internet hospitals, also helped resource-constrained regions heavily affected by the pandemic to be assisted remotely [14].

Technologies and AI were also helpful in enabling physical distancing: robots and uncrewed vehicles assisted in contact-free deliveries, including food and medical supplies [13,14].

### 3.3. National Strategy: Internet plus Healthcare

#### 3.3.1. Contexts

The unequal distribution of quality health resources has become a vital livelihood problem affecting people’s health in China [15]. In addition to income inequality, geographic distance is one factor that limits rural people’s access to quality health resources [16]. People living in rural areas with poor health services must travel long distances to find good hospitals/doctors in urban areas. This leads to overcrowding in some urban hospitals and longer waiting times. Healthcare reform in China to address the problem of healthcare in rural areas [17] has become a focus, since health is one of the most critical factors of equality.

China’s digital economy has proliferated since the end of the first decade of the 21st century, and China has a high rate of use of digital services [18]. Within this context, the Chinese government initiated the national strategy of IPHC to promote the use of the Internet in transforming, modernizing, and upgrading the healthcare industry [19]. One of the objectives of this national strategy is to address healthcare issues in rural areas.

#### 3.3.2. Implementation

IPHC integrates the Internet with the healthcare industry from all around. Its application includes health education, medical information queries, Electronic Health Records or Electronic Medical Records (EHR/EMRs), disease risk assessments, online disease consultations, electronic prescriptions, remote consultations, and various remote forms of health and medical services such as treatment and rehabilitation. Chinese hospitals use the Internet and mobile technologies to alleviate the challenges patients encounter when obtaining hospital services. Since the outbreak of COVID-19, IPHC has played a role in “high efficiency and low risk” healthcare delivery by enabling a more efficient pandemic response and launching a variety of valuable services such as addressing pandemic queries in real time, facilitating online consultations, and providing home isolation guidelines [20].

#### 3.3.3. Development

The originality of the design of the national strategy of IPHC can be traced back to the penetration of digital technologies across the healthcare industry over the past two decades (Table A2). Many practices have emerged that combine the Internet with healthcare services such as telemedicine, Internet diagnosis and treatment, and Internet hospitals.

After analyzing the commonalities and differences of these practices, we observed that the Internet hospital encompasses a healthcare delivery method that enables patients to be remotely examined by physicians with information communication technologies (Table 1).

Telemedicine is a new medical model formed with the development of computer technology and communication technology, which closely combines modern medicine with computer and communication technology. It can meet the needs of medical help or medical cooperation across hospitals, regions, and even countries, and realize the sharing of medical and health resources to the greatest extent. This medical model is of great value to alleviate the problems of “available”, “approachable”, and “affordable” healthcare in China. The development of telemedicine in China has experienced the stages of simple communication by telephone, communication software such as QQ and WeChat, professional video systems, and 4K ultra definition onsite video. However, even if the video telemedicine of onsite displays is realized by using advanced technology, the core data of medical diagnosis cannot be collected and transmitted in time.

In conclusion, telemedicine and Internet diagnosis and treatment are important ways for medical institutions to carry out Internet medicine. However, Internet hospitals can provide telemedicine, Internet diagnosis, and treatment and provide medical services that do not exceed the scope of diagnosis and treatment subjects of the entity medical institutions they rely on.

### 3.4. Internet Hospital

#### 3.4.1. Definition and Features

The Internet hospital has a fundamental feature of dependency on physical hospitals. There are two types of Internet hospitals: (1) physical healthcare institutions providing Internet hospital services can be registered as an Internet hospital at the same time; (2) independent Internet hospitals with medical services rely on physical medical entities, including hospitals jointly established by digital companies and physical hospitals.

**Figure 3:**
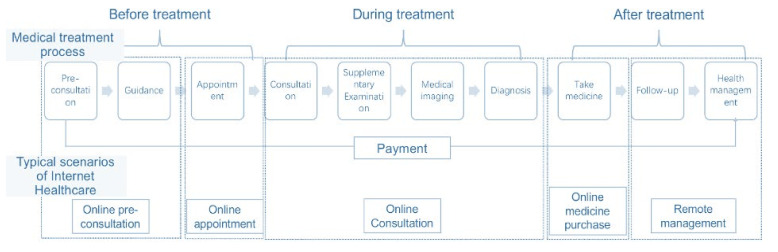
The medical treatment process in the physical hospital and online method. Adapted from source: Xinhua.net [20]).

The Internet hospital redefines the healthcare delivery concept and revolutionizes the traditional health sector [22]. This revolutionization happens in every corner of the industry, covering the whole treatment process, focusing on follow-up and routine consultation, as well as integrating inquiry, prescription, payment, and drug distribution (Figure 3).

#### 3.4.2. Functions and Role

The Internet hospital helps to alleviate the scarcity of quality healthcare resources in rural areas. It links medical resources and doctors from different physical hospitals. The developed regions with rich medical resources (mainly the eastern coastal regions) are equipped with a solid ability to independently establish Internet hospitals, which provide stable and sufficient funds for informatization and digitalization. In growing regions, mainly in Western and Central China, where medical resources are scarce, local primary-level healthcare institutions are usually connected with large hospitals to realize medical information exchange and sharing.

**Figure 4:**
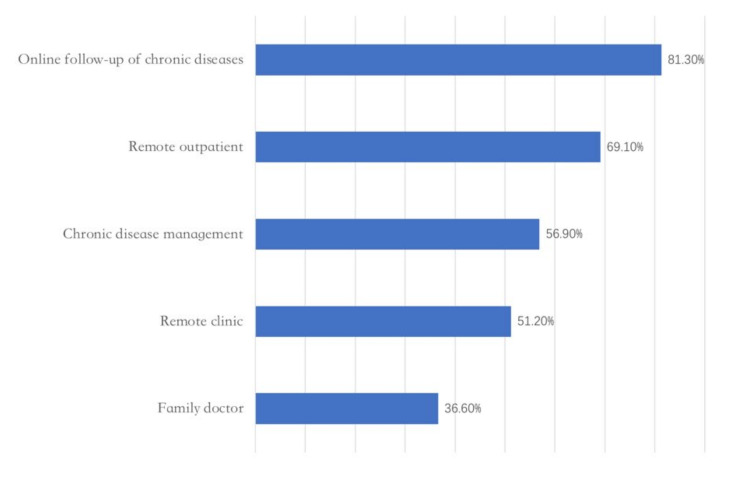
Core medical businesses. Adapted from source: National Telemedicine Center of China [23]).

In the core medical businesses (Figure 4), with the clarification of the business scope of Internet hospitals, the “online follow-up of chronic diseases”, as the essential company of Internet diagnosis and treatment and Internet hospitals, has a coverage rate of more than 80%. The popularization rate of “family doctor” (39%) services is relatively low, which needs further improvement.

**Figure 5:**
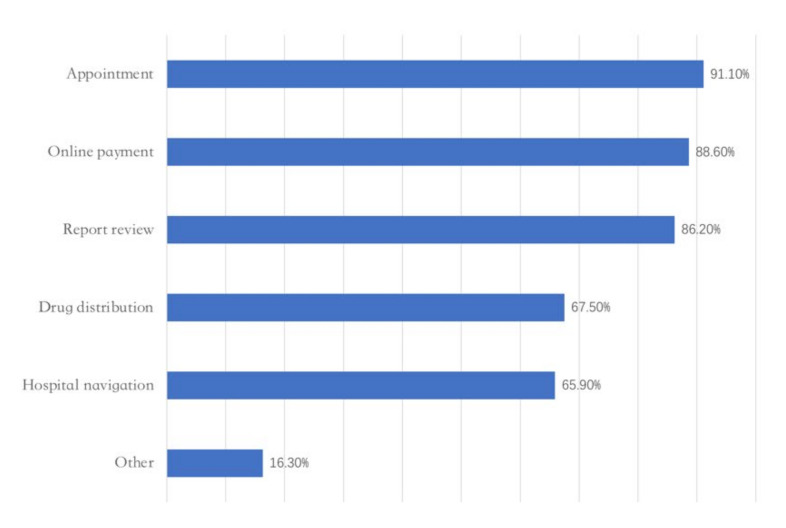
Regular services in hospitals. Adapted from source: National Telemedicine Center of China [23]).

**Figure 6:**
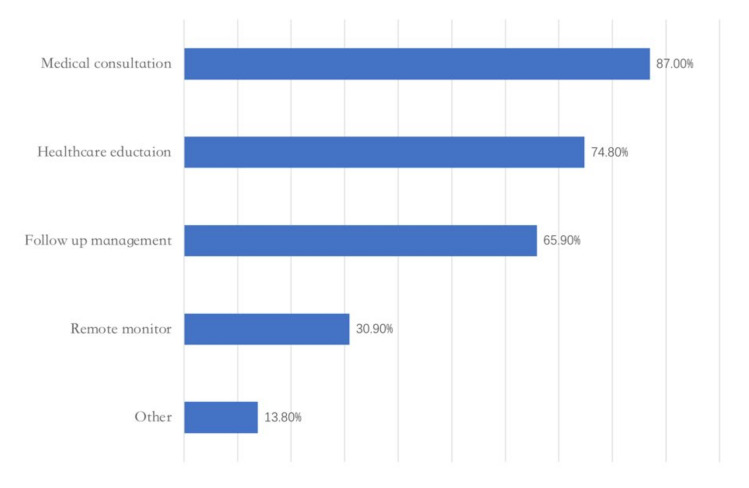
Non-core medical businesses. Adapted from source: National Telemedicine Center of China [23]).

At present, the “appointment” (90%), “online payment” (89%), and “report review” (86%) of regular services (Figure 5) and the “medical consultation” (87%) of non-core medical services are the most used services in Internet hospitals (Figure 6).

#### 3.4.3. Evolving Trend

The first Internet hospital was established in 2014 in Guangdong [24], which aroused discussion about its potential to transform the healthcare industry in China (Table A3). In December 2015, Guizhou province first issued the “Implementation Plan for the Pilot Work of Internet hospital” [25]. The plan discusses the service mode, working mechanism, policy system, and management methods. The period 2014–2016 witnessed an increase in Internet hospitals, while 2016–2018 experienced a downturn. On the one hand, many hospitals were at the stage of transformation while the policies were not clear; on the other hand, doctors and patients were worried about the Internet hospitals’ service quality and data safety [22].

**Figure 7:**
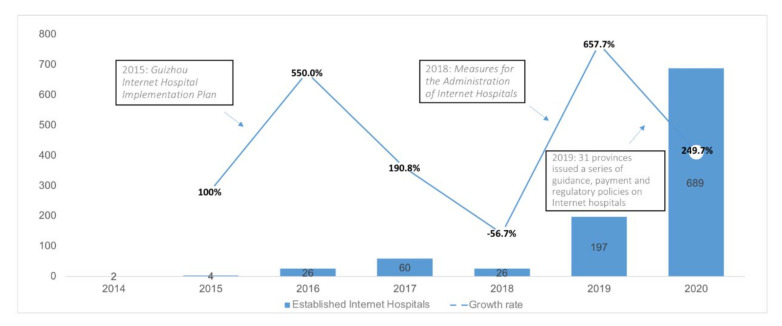
Policies issued by the government and established Internet hospitals in China, 2014–2019. Adapted from source: National Telemedicine Center of China [23]).

The establishment of the national strategy of Internet plus Healthcare in April 2018 gave momentum to the resurgence of Internet hospitals. Three important documents were released later in July 2021 by the National Health Commission (NHC): “Measures for the administration of Internet diagnosis and treatment (for Trial Implementation)”, “Measures for the administration of Internet hospitals (for Trial Implementation), and “Specification for telemedicine service management (for Trial Implementation)” [26]. These documents provide detailed regulations on the development of the Internet hospital and encourage local governments to issue implementation rules on guidance, payment, and regulations. In 2019, there were 197 Internet hospitals compared to 26 in 2018, showing a sharp increase. In 2020, that number was as high as 689 (Figure 7).

### 3.5. Internet Hospital and COVID-19

#### 3.5.1. Empowering the Response to COVID-19

COVID-19 is highly contagious, and large central hospitals have become a high-risk area for cross-infection. For patients with primary diseases who need to go to the hospital, if their condition is mild, they can choose an online consultation and Internet medical distribution to replace hospital consultation, which can significantly reduce the risk of infection and help alleviate the pressure of hospital reception. Driven by both user demands and policy incentives, both physical medical institutions and Internet medical enterprises have opened online fever clinics, Internet diagnosis and treatment platforms, and Internet hospitals [5]. The pandemic isolated the spatial distance between people, and it is Internet hospitals that can fill that space. Their advantages of high efficiency and low risk are more evident than ever before due to the pandemic.

Internet hospitals are at the front line of pandemic prevention and control. The NHC issued documents twice in four days, and various localities have also published several Internet hospital policies to encourage Internet diagnosis and treatment [5,21]. Among them, Guangdong, Shandong, Jiangsu, and other provinces released a list of Internet hospitals, and Hubei, Shanghai, Zhejiang, and other provinces launched official Internet diagnosis and treatment platforms.

#### 3.5.2. Encouraging Policies during COVID-19

The pandemic has dramatically promoted the demand for Internet hospitals, and the popularity of Internet hospitals is expected to increase significantly in the later stage of the pandemic. From offline to online, there has been a significant change in users’ medical habits under the influence of COVID-19. It is being realized that patients do not need to go to hospitals because of minor ailments and chronic diseases. Even the elderly have learned to buy medicine online. There is no doubt that COVID-19 has accelerated the penetration of Internet medical services on the client side.

In 2020, driven by the demand for pandemic prevention and control, local physical hospitals accelerated the construction pace. The NHC issued a series of documents on February 4 and February 7 [5], calling for the development of Internet diagnostic services, requiring all provincial health administrative departments to establish an Internet medical service platform to prevent and control the pandemic.

Noticing the strategic role of Internet hospitals in COVID-19 response and future medical reforms, the Chinese government is taking action to support their development. Pilot cities are implementing trial policies to solve the problem of insurance covering Internet hospitals.

## 4. Discussion

### 4.1. Lack of Efficient Operation and Management

*“Internet hospitals are not fully operational yet.”*

*—Dr. Sun, Gastroenterology Doctor of Traditional Chinese Medicine (TCM) in a second-tier hospital in Shanghai*

From Dr. Sun’s observation, due to the pandemic, the operation of Internet hospitals in 2020 is much better than that in 2019. There are around 50 online diagnoses and treatments per day. “Some patients are too busy to have time to come to the hospital, and their condition is not very serious.” In his gastroenterology department, the primary responsibility is the follow-up of medication for chronic patients, such as discomfort after colonoscopy or gastroscopy examination and consultation after long-term use of TCM. Internet medical treatment is limited to follow-up visits and general specialties. Thus, the efficiency of diagnosis and treatment is not high, the value of doctors is not effectively reflected, and the enthusiasm of doctors to participate is significantly reduced.

The four methods of diagnosis are general terms used by the doctors of the TCM for diagnosing illness, including diagnosis through observation, diagnosis through auscultation and olfaction, diagnosis through inquiry, and diagnosis through pulse feeling. With the help of Internet technology, online “face-to-face” diagnosis and treatment can also be realized. Through high-definition cameras, doctors can see the tongue coating, complexion, and expression of patients at the other end of the device. However, the time for online diagnosis and treatment is usually longer than offline. Dr. Sun said he could obtain a patient’s information in 3 to 5 min through offline communication, observation, and examination, but the information received online is more limited in the same period.

What is the role of TCM Internet hospitals in diagnosis and treatment? Some TCM Internet hospitals define themselves as a platform to provide intermediary services, only provide Internet technical help, do not participate in the communication between doctors and patients, and are not responsible for the source and correctness of the information released by doctors. However, the essence of a traditional Chinese medicine Internet hospital is that of a medical institution. According to the provisions of China’s law, medical institutions are the main body of medical responsibility. The hospital should manage and review the articles and comments published by doctors on its platform, and the information displayed is reviewed by the hospital by default. There are various theories and many schools of traditional Chinese medicine. Doctors have their own unique understanding of traditional Chinese medicine, and traditional Chinese medicine pays attention to adjusting measures to time, place, and individual conditions. It is difficult to formulate a generally accepted standard. However, the lack of standards makes it difficult to judge whether there is fault in diagnosis and treatment activities, which is not conducive to the electronic information of diagnosis and treatment activities of traditional Chinese medicine, and is not convenient for the collection, circulation, and use of medical Big Data. The difficulty of standardization of TCM is the main factor restricting the development of Internet hospitals and the industrialization of traditional Chinese medicine.

### 4.2. Lack of Standardized Service Process and Treatment Norms

*“There is a lack of unified standards for the expenses incurred in the diagnosis and treatment of Internet hospitals.”*

*—Dr. Liu, Deputy Director of Neurosurgery in a tertiary hospital in Beijing*

To ensure the safety of patients, according to regulations, hospitals cannot carry out Internet diagnosis and treatment activities for first-time patients. Dr. Liu shared his experience in the joint outpatient service of his hospital and a community hospital in Beijing. The patient’s examination in the community hospital can be uploaded to Dr. Liu’s computer through the online medical system. The community hospital can also use digital auscultation equipment to transmit the patient’s breathing sound to Dr. Liu in real time. After the patients have the joint outpatient records, they can make an online appointment with Dr. Liu for a follow-up visit through the Internet hospital.

The function of Internet hospitals is to reduce patients’ need to run errands. The inability to open the Internet for the first diagnosis has become a complex problem restricting the development of Internet hospitals. Dr. Liu also pointed out significant differences in the implementation standards of various hospitals. In some places, as long as patients upload their medical records, there are no restrictions on Internet hospital treatment. Nevertheless, some areas restrict only those patients initially diagnosed in their hospital to be followed up with via their Internet hospital system.

Another problem is unreasonable online charging. More cases need consultation with the increasing number of primary medical institutions joining the Internet hospital telemedicine union nationally. Dr. Liu and his colleagues said they were exhausted. There is no transparent pricing system for services provided by Internet hospitals. It is not clear who will pay or how much to pay for telemedicine services. Taking online follow-up as an example, Beijing charges RMB 50 (the highest charge in China), while the offline price of well-known experts ranges from RMB 100 to RMB 300. The cost of online and offline diagnosis and treatment is different, which frustrates the enthusiasm and initiative of doctors’ online visits and cannot reflect the value and differentiation of expert knowledge.

### 4.3. Lack of Effective Promotion and Well-Equipped System

*“Many patients do not have a strong willingness to visit the Internet hospital with a weak knowledge of Internet hospital and reimbursement policy.”*

*—Dr. Zhou, Deputy Director of Gastroenterology, working with Internet hospital in Shandong Province*

At present, publicity for Internet hospitals is not in place. Most patients do not realize the convenience of Internet hospitals or even know what kind of healthcare services Internet hospitals offer.

If patients from primary medical institutions in villages want to find doctors in major hospitals in Shandong Province for remote consultation, part of the expenses can be reimbursed, according to Dr. Zhou. If they are going to consult doctors from hospitals in Beijing or Shanghai, the exceeding cost will be covered by the exceptional financial support of the local government. However, the real difficulty is that many patients do not know the specific reimbursement policy well.

Dr. Zhou stressed the importance of high demands for digital devices for Internet hospitals. Limited by hardware equipment (including mobile phones, computers, etc.) and network environment, the patient symptoms observed by doctors may be inaccurate or distorted, which will affect diagnosis accuracy. Some older patients need the help of their families to log into their Internet hospital account successfully. Patients who visit the Internet hospital for the first time usually need to spend more time downloading digital applications and debugging equipment. If the patient uses the Internet hospital for the first time, he or she usually needs to spend more time downloading application software and debugging devices.

In recent years, the charging standards of Internet hospitals have been opaque, the service quality has been uneven, and the number of related adverse events has increased, reflecting the lack of risk management, which is an important risk source of medical innovation in China. The Internet medical model is very different from the traditional medical model. The different endpoints covered in the Internet medical model will produce a large amount of medical data. These data are the basis for data analysis, modeling, AI, and health management. At present, there is a lack of corresponding use specifications in the whole circulation process of data collection, storage, exchange, and processing. It is necessary to formulate clear policies and standards to reduce potential safety hazards, and achieve necessary informed consent. In addition, due to the weak information construction and lack of security measures in most hospitals, the medical equipment connected to the Internet is vulnerable to hacker attacks, and there are major security risks. On the one hand, medical equipment is often related to the personal safety of patients. On the other hand, when medical devices are connected to the hospital network system, hackers may steal private medical information.

## 5. Conclusions

Public hospitals will lead the development of Internet hospitals. The number of Chinese tier 1/2/3 hospitals continued to grow at mid-single-digit rates in the past three years and is expected to slow down as the government invests more resources to improve existing hospital capacity. Under the impact of COVID-19 in 2020, the offline business of medical institutions was restricted. The demand for online consultation and inquiry has multiplied. The change in supply and demand forced medical institutions, especially public hospitals, to take the initiative to launch Internet hospitals. By the end of 2020, the number of Internet hospitals reached 1004. Among them, public hospitals play a dominant role, accounting for nearly 70%. Public hospitals have a good reputation in China, and the treatment habits of Chinese patients indicate that they still tend to choose public hospitals. In terms of demand, Internet hospitals are far from satisfactory, and there will be a greater demand at the later stage. With the improvement of national assessment standards for public hospitals in information and digitalization construction, Internet hospitals will also become a necessary standard to measure the medical quality of a physical hospital.

Different tiers of Internet hospitals will have differentiated development. The essence of Internet hospital development is medical treatment. Internet technology is a tool to link doctors and patients, and doctors and doctors. Patient services, payment, health, and disease management will be transferred online. Patient services such as guidance, registration, examination, operation, medication, and patient follow-up management should be coordinated in Internet hospitals.

Hospitals at different levels have different functional positioning, and the scope of setting up Internet hospitals cannot cross the border. The first-tier hospitals are still growing in scale and continuing to develop capacity for acute illness treatment, e.g., oncology and cardiovascular disease. The second-tier hospitals are mainly focused on quality improvements. The tertiary hospital has substantial growth in its capacity, driven by direct government investments. Different types of Internet hospital business also have differences. For example, gynecological Internet hospitals cannot provide surgical consultations. With the help of the Internet, doctors in different hospitals can work in groups to jointly manage patients. Dr. Liu thinks, in the future, patients who come to large hospitals for surgery will return to primary medical institutions for rehabilitation. Doctors will carry out healthcare coordination inside and outside the hospital around patient disease treatment, performing their own functions.

Internet hospitals expand influence in chronic disease management. According to the National Bureau of Statistics (NBS), the number of individuals more than 65 years old reached 176.0 million in 2019, which accounted for 12.6% of the total population. This population is expected to continue its growth momentum in the future. Moreover, the prevalence of chronic health problems, such as cardiovascular disease, grew over the past decade and is expected to continue to grow significantly in the future due to lifestyle changes. The aging trend and changing disease patterns will create massive demand for medical services in China. The popularity of health-oriented digital medical services is increasing, and Internet hospitals are developing rapidly with a chronic disease management model. New telemedicine-promoting policies and ubiquitous mobile phone access in China now raise the possibility that telemedicine could help bridge gaps in care for chronic medical conditions. Even after COVID-19 is controlled, Internet hospitals have the potential to address persistent obstacles to primary healthcare, including the scarcity of trained healthcare workers, the difficulty of patient transportation, and in-person care costs.

The pandemic has exposed that there is still room for improvement in hospital information construction, and the promotion of Internet hospitals has also put forward higher requirements for the Internet hospital system. Users’ acceptance of online services, the online process of hospitals, and the degree of policy support for the Internet medical industry all showed breakthrough progress. In the past, Internet healthcare participants were Internet companies with technological advantages. With the impact of increased policy promotion and the gradual formation of user habits, the hospital will gradually extend traditional services to online consultation, follow-up of chronic and common diseases, and other businesses, and gradually take a central role in the Internet medical industry, which is also in line with the direction of policy supervision.

Finally, the limitations of this research are acknowledged; furthermore, the generalizability of the findings may be limited. As a survey of Internet hospitals in China, the sample size of our qualitative research is insufficient because some areas with few hospitals are not included, such as Xinjiang and Tibet. Therefore, the research scope needs to be further expanded. In addition, we only take Chinese public hospitals as the survey object, which is insufficient in reflecting the universality of Internet online services during the pandemic period. Enterprise-led Internet hospitals are also essential participants, which will be the focus of future research.

## Figures and Tables

**Table 1:** Comparison of different Internet-integrated healthcare practices [5,21].

	Policy	Actor	Service	Treatment
Telemedicine	Specifications for the Administration of Remote Medical Services (for Trial Implementation) July 2011	Qualified medical institution relying on physical hospitals (only carried out between medical institutions)	Remote diagnostic imaging, remote monitoring guidance, remote surgical guidance	All kinds of diseases
Internet diagnosis and treatment	Measures for the Administration of Internet hospitals (for Trial Implementation) July 2018	Qualified doctors	Medical consultation	Follow-up services for chronic diseases
Internet hospital	Article 2 of the Measures for the Administration of Internet hospitals (for Trial Implementation) July 2018	Qualified medical institution relying on physical hospitals	Medical consultation, integrating inquiry, prescription, payment, and drug distribution	Follow-up of chronic diseases and some common diseases

## Data Availability

Not applicable.

## Appendix A

**Table A1:** List of interviewees (anonymized).

	Date of the Interview	Region	Gender	Major
Resident doctor1	9 November 2021	Shanghai	Male	Chinese traditional therapy
Deputy chief doctor 1	10 November 2021	Beijing	Male	Neurosurgery
Deputy chief doctor 2	13 November 2021	Shandong	Male	Gastroenterology

## Appendix B

**Table A2:** Internet hospital policies issued during COVID-19 (national level) [5].

Issuing Body	Issuing Date	Subject	Statement
NHC	4 February 2020	Notice on applying information technologies to support the prevention and control of COVID-19	Registered and approved Internet hospitals and their links should be published on the website of the local health commission. Encouraging medical institutions to carry out online voluntary counseling and guidance for COVID-19. Encouraging online return visits and drug delivery services for chronic diseases in order to reduce the incidence of cross-infection offline.
NHC, NHSA	2 March 2020	Guiding Opinions on the provision and reimbursement of online health services during the COVID-19 outbreak	Reimburse client-to-provider telemedicine for common illness and follow-up visits for non-communicable diseases offered by accredited Internet hospitals.

**Table A3:** Internet hospital policies issued during COVID-19 (provincial level) [5,8,16,20].

Issuing Body	Issuing Date	Statement
Anhui Health Commission	24 January 2020	Five Internet hospitals for urgent fever outpatient service, with senior experts providing consultation services for fever patients online.
Shandong Health Commission	27 January 2020	The first batch of 48 Internet hospitals opened for online consultation.
Hubei Health Commission	1 February 2020	Two Internet hospitals for online fever outpatient service, providing online consultation.
Guangdong Health Commission	1 February 2020	The first branch of 57 Internet hospitals opened for fever outpatient service and consultation.
Zhejiang Health Commission	3 February 2020	Opened Internet hospital COVID-19 channel, which was replied to by respiratory physicians from hospitals across the province online.
Jiangsu Health Commission	6 February 2020	41 hospitals for fever outpatient consultation services.
Tianjin Health Commission	28 February 2020	Rapid approval of Internet hospital diagnosis and treatment projects to alleviate the pressure of offline clinics.
